# Crystal structure of bis­{μ-1-[(*E*)-(3-meth­oxy­phen­yl)diazen­yl]naphthalen-2-olato-κ^3^ *N* ^2^,*O*:*O*}bis­({1-[(*E*)-(3-meth­oxy­phen­yl)diazen­yl]naphthalen-2-olato-κ^2^ *N* ^2^,*O*}copper(II))

**DOI:** 10.1107/S2056989015020824

**Published:** 2015-11-07

**Authors:** Souheyla Chetioui, Noudjoud Hamdouni, Christian G. Bochet, Jean-Pierre Djukic, Corinne Bailly

**Affiliations:** a Unité de Recherche de Chimie de l’Environnement et Moléculaire Structurale, (CHEMS), Faculté des Sciences Exactes, Département de Chimie, Université Constantine 1, Constantine 25000, Algeria; b Laboratoire de Cristallographie, Département de Physique, Université Constantine 1, Constantine 25000, Algeria; c Chemistry Department, University of Fribourg, Chemin du Musee 9, CH-1700 Fribourg, Switzerland; d Laboratoire de Chimie et Systémique Organométallique (LCSOM), Institut de Chimie, Université de Strasbourg, UMR 7177, F-67070 Strasbourg Cedex, France; e Service de Radiocristallographie, Institut de Chimie, Université de Strasbourg, UMR 7177, 67008 Strasbourg Cedex, France

**Keywords:** crystal structure, dinuclear Cu complex, azo dyes, hydrogen bonding

## Abstract

The title dinuclear Cu^II^ complex, [Cu_2_(C_17_H_13_N_2_O_2_)_4_], is located on an inversion centre. The Cu^II^ atoms are each five-coordinated in a distorted square-pyramidal geometry by two N atoms and two O atoms from two bidentate ligands and one bridging O atom from another ligand. In the dinuclear complex, the Cu⋯Cu separation is 3.366 (3) Å. In the crystal, complex mol­ecules are linked *via* weak C—H⋯O hydrogen bonds, forming a layer parallel to (-101).

## Related literature   

For general background to azo compounds and their use in dyes, pigments and advanced materials, see: Lee *et al.* (2004 ▸). For background to metal–azo complexes, see: Carella *et al.* (2007 ▸); Kulikovska *et al.* (2007 ▸); Patnaik *et al.* (2007 ▸); Leng *et al.* (2001 ▸). For related structures of azo compounds, see: Chetioui *et al.* (2013*a* ▸,*b* ▸).
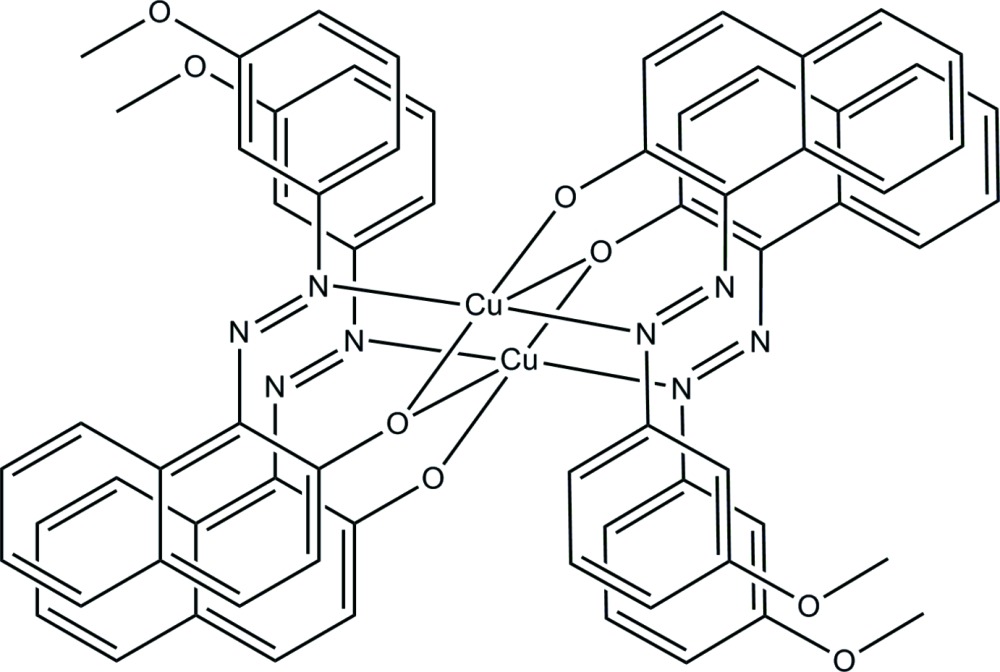


## Experimental   

### Crystal data  

[Cu_2_(C_17_H_13_N_2_O_2_)_4_]
*M* *_r_* = 1236.26
Monoclinic, 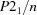

*a* = 16.260 (5) Å
*b* = 7.707 (5) Å
*c* = 22.325 (5) Å
β = 104.268 (5)°
*V* = 2711 (2) Å^3^
*Z* = 2
Mo *K*α radiation
μ = 0.86 mm^−1^
*T* = 173 K
0.45 × 0.10 × 0.04 mm

### Data collection  

Bruker APEXII CCD diffractometer
Absorption correction: multi-scan (*SADABS*; Bruker, 2006 ▸) *T*_min_ = 0.855, *T*_max_ = 0.966
25289 measured reflections
6516 independent reflections
4941 reflections with *I* > 2σ(*I*)
*R*_int_ = 0.041

### Refinement  

*R*[*F* ^2^ > 2σ(*F* ^2^)] = 0.040
*wR*(*F* ^2^) = 0.091
*S* = 1.02
6516 reflections
388 parameters
H-atom parameters constrained
Δρ_max_ = 0.46 e Å^−3^
Δρ_min_ = −0.33 e Å^−3^

### 

Data collection: *APEX2* (Bruker, 2006 ▸); cell refinement: *SAINT* (Bruker, 2006 ▸); data reduction: *SAINT*; program(s) used to solve structure: *SIR92* (Altomare *et al.*, 1993 ▸); program(s) used to refine structure: *SHELXL2014* (Sheldrick, 2015 ▸); molecular graphics: *ORTEP-3 for Windows* (Farrugia, 2012 ▸); software used to prepare material for publication: *WinGX* (Farrugia, 2012 ▸).

## Supplementary Material

Crystal structure: contains datablock(s) global, I. DOI: 10.1107/S2056989015020824/is5425sup1.cif

**Fig. 1.**
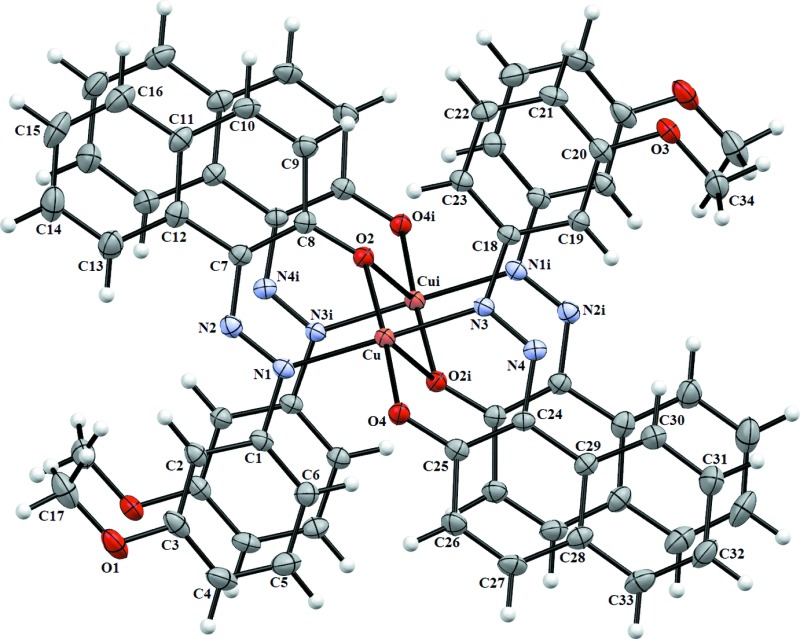
The molecular structure of the title compound, with atom labels and 50% probability displacement ellipsoids for non-H atoms. [Symmetry code: (i) −x + 1, −y, −z + 2.]

Structure factors: contains datablock(s) I. DOI: 10.1107/S2056989015020824/is5425Isup2.hkl

x y z . DOI: 10.1107/S2056989015020824/is5425fig1.tif
The mol­ecular structure of the title compound, with atom labels and 50% probability displacement ellipsoids for non-H atoms. [Symmetry code: (i) −*x* + 1, −*y*, −*z* + 2.]

**Fig. 2.**
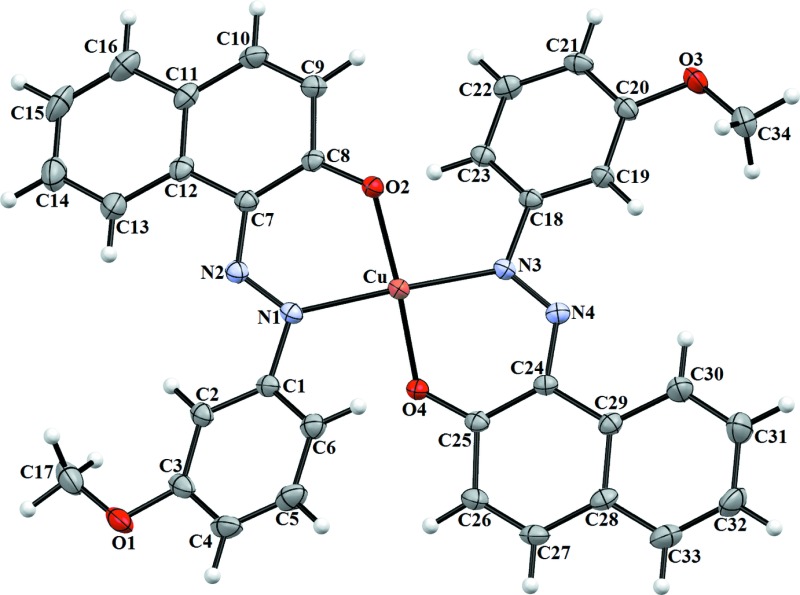
Asymmetric unit of the title compound.

**Fig. 3.**
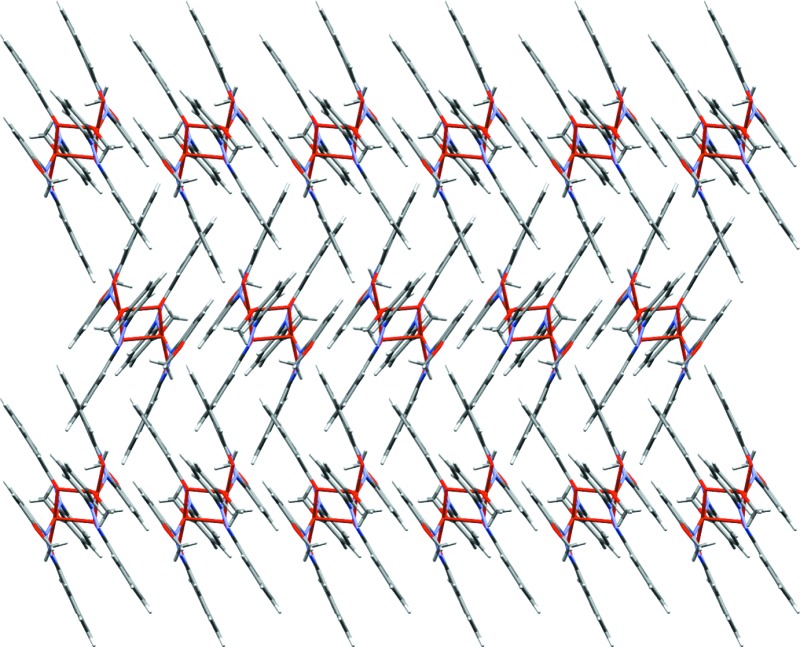
A packing diagram of the title compound viewed along the a axis.

. DOI: 10.1107/S2056989015020824/is5425fig2.tif
Asymmetric unit of the title compound.

a . DOI: 10.1107/S2056989015020824/is5425fig3.tif
A packing diagram of the title compound viewed along the *a* axis.

CCDC reference: 1434923

Additional supporting information:  crystallographic information; 3D view; checkCIF report

## Figures and Tables

**Table 1:** Hydrogen-bond geometry (Å, °)

*D*—H⋯*A*	*D*—H	H⋯*A*	*D*⋯*A*	*D*—H⋯*A*
C22—H22⋯O4^i^	0.93	2.45	3.267 (3)	146
C32—H32⋯O1^ii^	0.93	2.51	3.394 (4)	158

Symmetry codes: (i) 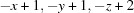; (ii) 
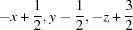
.

## supplementary crystallographic information

### S1. Comment

Azo compounds are very important in the fields of dyes, pigments and advanced materials (Lee *et al.*, 2004). Azo dyes are synthetic colours that contain an azo group, as part of the structure. They are characterized by the azo linkage (–N=N–). We are involved in the color generation mechanism of azo pigments typically characterized by the chromophore of the azo group (–N=N–) (Chetioui *et al.*, 2013*a*,*b*) to synthesize new copper complex with Cu(OAc)_2_·H_2_O. Metal complexes with azo ligands show interesting chemical and physical properties and are of interest as new materials, for example in bioinorganic and coordination chemistry, as well as in biological systems which can lead to the development of new products with specific properties (Carella *et al.*, 2007; Kulikovska *et al.*, 2007; Patnaik *et al.*, 2007; Leng *et al.*, 2001). In this work the structure of the title molecule, Cu_2_(C_17_H_13_N_2_O_2_)_4_, is reported.

The title dicopper complex (Fig. 1) consists of two inversion related asymmetric units (Fig. 2), in which the Cu^II^ atoms are each coordinated by two *N*,*O*-bidentate phenylazo-naphtholate ligands. The two N atoms and two O atoms around the Cu atom are *trans* to each other with an O2—Cu—N1 bond angle of 86.83 (7)° and an O2—Cu—N3 angle of 96.06 (7)°. The inversion related asymmetric units are linked by one bridging O atom [O2^i^; symmetry code: (i) −*x* + 1, −*y*, −*z* + 2] with O2^i^—Cu—O4 and O2—Cu—O2^i^ angles of 104.51 (6) and 81.69 (5)°, respectively, to form a distorted square-pyramidal geometry. In the crystal, molecules are linked *via* weak C—H···O hydrogen bonds (Table 1), forming a layer parallel to (101) (Fig. 3).

### S2. Experimental

The title compound was synthesized by the following procedures: (*E*)-1-[(3-methoxyphenyl)diazenyl]naphthalen-2-ol (0.55 g, 2.0 mmol) and Cu(O*Ac*)_2_·H_2_O (0.20 g, 1.0 mmol) was stirred at 298 K in the methanol (10 ml) for 48 h. Volatile materials were removed under vacuum and the residue was washed twice from hexane solution to give red solids. The resulting solids were crystallized from CH_2_Cl_2_ to yield red crystals.

### S3. Refinement

H atoms were included in calculated positions with C—H = 0.93 or 0.96 Å and were refined as riding with *U*_iso_(H) = 1.2*U*_eq_(C) or 1.5*U*_eq_(methyl C).

### Crystal data

**Table:**

[Cu_2_(C_17_H_13_N_2_O_2_)_4_]	*F*(000) = 1276
*M**_r_* = 1236.26	*D*_x_ = 1.514 Mg m^−^^3^
Monoclinic, *P*2_1_/*n*	Mo *K*α radiation, λ = 0.71073 Å
Hall symbol: -P 2yn	Cell parameters from 2052 reflections
*a* = 16.260 (5) Å	θ = 3.1–28.6°
*b* = 7.707 (5) Å	µ = 0.86 mm^−^^1^
*c* = 22.325 (5) Å	*T* = 173 K
β = 104.268 (5)°	Plate, red
*V* = 2711 (2) Å^3^	0.45 × 0.10 × 0.04 mm
*Z* = 2	

### Data collection

**Table:**

Bruker APEXII CCD diffractometer	6516 independent reflections
Radiation source: fine-focus sealed tube	4941 reflections with *I* > 2σ(*I*)
Triumph monochromator	*R*_int_ = 0.041
φ and ω scans	θ_max_ = 28.0°, θ_min_ = 1.8°
Absorption correction: multi-scan (*SADABS*; Bruker, 2006)	*h* = −21→21
*T*_min_ = 0.855, *T*_max_ = 0.966	*k* = −4→10
25289 measured reflections	*l* = −28→29

### Refinement

**Table:**

Refinement on *F*^2^	Primary atom site location: structure-invariant direct methods
Least-squares matrix: full	Secondary atom site location: difference Fourier map
*R*[*F*^2^ > 2σ(*F*^2^)] = 0.040	Hydrogen site location: inferred from neighbouring sites
*wR*(*F*^2^) = 0.091	H-atom parameters constrained
*S* = 1.02	*w* = 1/[σ^2^(*F*_o_^2^) + (0.0392*P*)^2^ + 1.8473*P*] where *P* = (*F*_o_^2^ + 2*F*_c_^2^)/3
6516 reflections	(Δ/σ)_max_ = 0.001
388 parameters	Δρ_max_ = 0.46 e Å^−^^3^
0 restraints	Δρ_min_ = −0.33 e Å^−^^3^
0 constraints	

### Special details

**Table:**

Geometry. Bond distances, angles *etc*. have been calculated using the rounded fractional coordinates. All su's are estimated from the variances of the (full) variance-covariance matrix. The cell e.s.d.'s are taken into account in the estimation of distances, angles and torsion angles
Refinement. Refinement of *F*^2^ against ALL reflections. The weighted *R*-factor *wR* and goodness of fit *S* are based on *F*^2^, conventional *R*-factors *R* are based on *F*, with *F* set to zero for negative *F*^2^. The threshold expression of *F*^2^ > σ(*F*^2^) is used only for calculating *R*-factors(gt) *etc*. and is not relevant to the choice of reflections for refinement. *R*-factors based on *F*^2^ are statistically about twice as large as those based on *F*, and *R*- factors based on ALL data will be even larger.

### Fractional atomic coordinates and isotropic or equivalent isotropic displacement parameters (Å^2^)

**Table:**

	*x*	*y*	*z*	*U*_iso_*/*U*_eq_	
Cu	0.43895 (2)	0.16467 (3)	0.96464 (2)	0.0168 (1)	
O1	0.03530 (9)	0.1354 (2)	0.96483 (8)	0.0327 (5)	
O2	0.51647 (8)	0.14161 (18)	1.04496 (7)	0.0179 (4)	
O3	0.83178 (9)	0.3080 (2)	0.93754 (8)	0.0298 (5)	
O4	0.35788 (9)	0.21050 (19)	0.88861 (7)	0.0197 (4)	
N1	0.34448 (10)	0.1210 (2)	1.00735 (8)	0.0168 (5)	
N2	0.34504 (10)	0.1706 (2)	1.06202 (8)	0.0184 (5)	
N3	0.52791 (10)	0.2345 (2)	0.91924 (8)	0.0164 (5)	
N4	0.52351 (11)	0.1912 (2)	0.86321 (8)	0.0177 (5)	
C1	0.26175 (13)	0.0628 (3)	0.97394 (10)	0.0180 (6)	
C2	0.18925 (13)	0.1284 (3)	0.98921 (10)	0.0198 (6)	
C3	0.11017 (13)	0.0752 (3)	0.95503 (11)	0.0255 (7)	
C4	0.10379 (15)	−0.0434 (3)	0.90725 (11)	0.0312 (8)	
C5	0.17587 (16)	−0.1075 (3)	0.89314 (11)	0.0298 (7)	
C6	0.25590 (14)	−0.0540 (3)	0.92616 (10)	0.0224 (7)	
C7	0.41663 (13)	0.2373 (3)	1.10110 (10)	0.0181 (6)	
C8	0.49944 (13)	0.2220 (3)	1.09202 (10)	0.0169 (6)	
C9	0.56755 (14)	0.2969 (3)	1.13794 (10)	0.0219 (6)	
C10	0.55333 (14)	0.3848 (3)	1.18703 (11)	0.0246 (7)	
C11	0.47097 (14)	0.4023 (3)	1.19736 (10)	0.0227 (6)	
C12	0.40167 (14)	0.3241 (3)	1.15493 (10)	0.0216 (6)	
C13	0.32039 (15)	0.3378 (3)	1.16662 (11)	0.0301 (7)	
C14	0.30890 (17)	0.4270 (4)	1.21729 (12)	0.0378 (9)	
C15	0.37733 (18)	0.5083 (4)	1.25783 (12)	0.0382 (9)	
C16	0.45651 (17)	0.4960 (3)	1.24817 (11)	0.0326 (8)	
C17	0.03923 (16)	0.2459 (4)	1.01626 (13)	0.0375 (9)	
C18	0.60985 (13)	0.3057 (3)	0.94875 (10)	0.0169 (6)	
C19	0.68012 (13)	0.2692 (3)	0.92585 (10)	0.0191 (6)	
C20	0.75860 (13)	0.3374 (3)	0.95597 (10)	0.0209 (6)	
C21	0.76648 (14)	0.4400 (3)	1.00807 (11)	0.0223 (6)	
C22	0.69622 (13)	0.4775 (3)	1.02969 (10)	0.0218 (6)	
C23	0.61679 (13)	0.4110 (3)	1.00022 (10)	0.0185 (6)	
C24	0.44976 (13)	0.1318 (2)	0.82480 (10)	0.0168 (6)	
C25	0.36914 (12)	0.1495 (3)	0.83733 (10)	0.0174 (6)	
C26	0.29605 (14)	0.1012 (3)	0.78953 (10)	0.0235 (7)	
C27	0.30412 (14)	0.0283 (3)	0.73592 (10)	0.0245 (7)	
C28	0.38469 (14)	0.0045 (3)	0.72267 (10)	0.0215 (6)	
C29	0.45840 (13)	0.0612 (3)	0.76664 (10)	0.0187 (6)	
C30	0.53741 (14)	0.0439 (3)	0.75208 (11)	0.0269 (7)	
C31	0.54277 (16)	−0.0265 (3)	0.69672 (12)	0.0343 (8)	
C32	0.47031 (16)	−0.0870 (3)	0.65411 (12)	0.0339 (8)	
C33	0.39286 (16)	−0.0727 (3)	0.66692 (11)	0.0294 (7)	
C34	0.82769 (15)	0.1918 (3)	0.88753 (12)	0.0325 (8)	
H2	0.30450	−0.09610	0.91630	0.0270*	
H3	0.17100	−0.18740	0.86120	0.0360*	
H4	0.05060	−0.07980	0.88470	0.0370*	
H6	0.19410	0.20630	1.02170	0.0240*	
H9	0.62290	0.28490	1.13400	0.0260*	
H10	0.59910	0.43550	1.21490	0.0300*	
H13	0.27410	0.28580	1.13970	0.0360*	
H14	0.25510	0.43340	1.22460	0.0450*	
H15	0.36870	0.57080	1.29140	0.0460*	
H16	0.50180	0.55010	1.27540	0.0390*	
H17A	−0.01720	0.27850	1.01780	0.0560*	
H17B	0.06640	0.18600	1.05360	0.0560*	
H17C	0.07120	0.34810	1.01220	0.0560*	
H19	0.67470	0.20030	0.89090	0.0230*	
H21	0.81930	0.48350	1.02840	0.0270*	
H22	0.70180	0.54800	1.06430	0.0260*	
H23	0.56930	0.43680	1.01480	0.0220*	
H26	0.24210	0.12030	0.79540	0.0280*	
H27	0.25560	−0.00710	0.70690	0.0290*	
H30	0.58650	0.08050	0.78030	0.0320*	
H31	0.59530	−0.03410	0.68740	0.0410*	
H32	0.47500	−0.13690	0.61720	0.0410*	
H33	0.34490	−0.11420	0.63870	0.0350*	
H34A	0.88270	0.18260	0.87920	0.0490*	
H34B	0.80980	0.07960	0.89810	0.0490*	
H34C	0.78780	0.23480	0.85150	0.0490*	

### Atomic displacement parameters (Å^2^)

**Table:**

	*U*^11^	*U*^22^	*U*^33^	*U*^12^	*U*^13^	*U*^23^
Cu	0.0133 (1)	0.0211 (1)	0.0158 (1)	−0.0002 (1)	0.0031 (1)	−0.0003 (1)
O1	0.0145 (7)	0.0488 (11)	0.0344 (10)	−0.0003 (7)	0.0051 (7)	0.0115 (8)
O2	0.0150 (7)	0.0213 (7)	0.0169 (8)	0.0020 (6)	0.0028 (6)	−0.0012 (6)
O3	0.0157 (7)	0.0429 (10)	0.0325 (10)	−0.0056 (7)	0.0090 (7)	−0.0074 (8)
O4	0.0156 (7)	0.0272 (8)	0.0155 (8)	0.0031 (6)	0.0026 (6)	−0.0003 (6)
N1	0.0142 (8)	0.0169 (8)	0.0185 (9)	0.0008 (6)	0.0025 (7)	0.0013 (7)
N2	0.0163 (8)	0.0208 (8)	0.0178 (9)	0.0007 (7)	0.0038 (7)	0.0028 (8)
N3	0.0134 (8)	0.0183 (8)	0.0168 (9)	−0.0015 (7)	0.0026 (7)	−0.0004 (7)
N4	0.0174 (8)	0.0179 (8)	0.0168 (9)	−0.0006 (7)	0.0026 (7)	−0.0006 (7)
C1	0.0170 (10)	0.0188 (10)	0.0172 (11)	−0.0033 (8)	0.0023 (8)	0.0042 (8)
C2	0.0176 (10)	0.0217 (11)	0.0204 (11)	−0.0010 (8)	0.0051 (8)	0.0049 (8)
C3	0.0163 (10)	0.0344 (13)	0.0246 (12)	−0.0020 (9)	0.0030 (9)	0.0127 (10)
C4	0.0235 (12)	0.0421 (14)	0.0236 (13)	−0.0137 (10)	−0.0024 (10)	0.0079 (11)
C5	0.0353 (13)	0.0297 (12)	0.0221 (13)	−0.0104 (10)	0.0028 (10)	−0.0024 (10)
C6	0.0235 (11)	0.0231 (11)	0.0206 (12)	−0.0024 (9)	0.0053 (9)	0.0016 (9)
C7	0.0173 (10)	0.0184 (10)	0.0178 (11)	0.0014 (8)	0.0028 (8)	0.0015 (8)
C8	0.0189 (10)	0.0164 (9)	0.0150 (10)	0.0002 (8)	0.0033 (8)	0.0033 (8)
C9	0.0186 (10)	0.0252 (11)	0.0200 (12)	−0.0002 (8)	0.0014 (8)	0.0003 (9)
C10	0.0244 (11)	0.0270 (11)	0.0192 (12)	−0.0046 (9)	−0.0007 (9)	−0.0018 (9)
C11	0.0301 (12)	0.0215 (10)	0.0166 (11)	0.0021 (9)	0.0057 (9)	0.0018 (9)
C12	0.0248 (11)	0.0221 (10)	0.0178 (11)	0.0050 (9)	0.0053 (8)	0.0044 (9)
C13	0.0279 (12)	0.0411 (13)	0.0218 (12)	0.0042 (11)	0.0073 (9)	0.0011 (11)
C14	0.0353 (14)	0.0505 (16)	0.0321 (15)	0.0114 (12)	0.0169 (12)	0.0013 (13)
C15	0.0564 (17)	0.0413 (15)	0.0209 (13)	0.0058 (13)	0.0173 (12)	−0.0045 (11)
C16	0.0444 (15)	0.0321 (13)	0.0208 (13)	−0.0015 (11)	0.0069 (11)	−0.0040 (10)
C17	0.0221 (12)	0.0458 (15)	0.0481 (17)	0.0037 (11)	0.0155 (11)	0.0111 (14)
C18	0.0152 (9)	0.0163 (10)	0.0183 (11)	−0.0017 (7)	0.0022 (8)	0.0019 (8)
C19	0.0180 (10)	0.0204 (10)	0.0186 (11)	−0.0027 (8)	0.0042 (8)	−0.0009 (8)
C20	0.0154 (9)	0.0231 (10)	0.0247 (12)	−0.0011 (9)	0.0057 (8)	0.0040 (9)
C21	0.0182 (10)	0.0207 (10)	0.0249 (12)	−0.0056 (8)	−0.0004 (9)	0.0017 (9)
C22	0.0256 (11)	0.0165 (10)	0.0219 (12)	−0.0016 (8)	0.0033 (9)	−0.0024 (9)
C23	0.0171 (10)	0.0174 (9)	0.0201 (11)	0.0008 (8)	0.0031 (8)	0.0009 (8)
C24	0.0168 (9)	0.0158 (10)	0.0166 (10)	0.0001 (7)	0.0019 (8)	0.0004 (8)
C25	0.0169 (9)	0.0162 (9)	0.0177 (11)	0.0016 (8)	0.0018 (8)	0.0039 (8)
C26	0.0161 (10)	0.0305 (12)	0.0219 (12)	0.0017 (9)	0.0007 (9)	0.0022 (10)
C27	0.0191 (10)	0.0291 (12)	0.0209 (12)	−0.0008 (9)	−0.0037 (9)	0.0015 (9)
C28	0.0253 (11)	0.0206 (10)	0.0162 (11)	0.0013 (9)	0.0008 (9)	0.0024 (8)
C29	0.0209 (10)	0.0177 (10)	0.0170 (11)	0.0014 (8)	0.0035 (8)	0.0015 (8)
C30	0.0231 (11)	0.0330 (12)	0.0243 (13)	−0.0026 (10)	0.0054 (9)	−0.0060 (10)
C31	0.0297 (13)	0.0430 (15)	0.0323 (14)	0.0017 (11)	0.0119 (11)	−0.0098 (12)
C32	0.0402 (14)	0.0423 (14)	0.0193 (13)	0.0058 (12)	0.0073 (11)	−0.0092 (11)
C33	0.0326 (13)	0.0314 (13)	0.0197 (12)	0.0021 (10)	−0.0022 (10)	−0.0031 (10)
C34	0.0233 (12)	0.0455 (15)	0.0313 (14)	0.0008 (10)	0.0119 (10)	−0.0005 (11)

### Geometric parameters (Å, º)

**Table:**

Cu—O2	1.929 (2)	C22—C23	1.395 (3)
Cu—O4	1.908 (2)	C24—C25	1.413 (3)
Cu—N1	2.026 (2)	C24—C29	1.446 (3)
Cu—N3	2.033 (2)	C25—C26	1.437 (3)
Cu—O2^i^	2.494 (2)	C26—C27	1.358 (3)
O1—C3	1.370 (3)	C27—C28	1.424 (3)
O1—C17	1.418 (3)	C28—C29	1.418 (3)
O2—C8	1.307 (3)	C28—C33	1.415 (3)
O3—C20	1.370 (3)	C29—C30	1.407 (3)
O3—C34	1.420 (3)	C30—C31	1.372 (4)
O4—C25	1.292 (3)	C31—C32	1.399 (4)
N1—N2	1.277 (3)	C32—C33	1.363 (4)
N1—C1	1.440 (3)	C2—H6	0.9300
N2—C7	1.371 (3)	C4—H4	0.9300
N3—N4	1.280 (3)	C5—H3	0.9300
N3—C18	1.441 (3)	C6—H2	0.9300
N4—C24	1.370 (3)	C9—H9	0.9300
C1—C2	1.400 (3)	C10—H10	0.9300
C1—C6	1.381 (3)	C13—H13	0.9300
C2—C3	1.386 (3)	C14—H14	0.9300
C3—C4	1.389 (3)	C15—H15	0.9300
C4—C5	1.378 (4)	C16—H16	0.9300
C5—C6	1.391 (4)	C17—H17A	0.9600
C7—C8	1.415 (3)	C17—H17B	0.9600
C7—C12	1.447 (3)	C17—H17C	0.9600
C8—C9	1.432 (3)	C19—H19	0.9300
C9—C10	1.356 (3)	C21—H21	0.9300
C10—C11	1.420 (3)	C22—H22	0.9300
C11—C12	1.416 (3)	C23—H23	0.9300
C11—C16	1.412 (3)	C26—H26	0.9300
C12—C13	1.413 (4)	C27—H27	0.9300
C13—C14	1.375 (4)	C30—H30	0.9300
C14—C15	1.398 (4)	C31—H31	0.9300
C15—C16	1.360 (4)	C32—H32	0.9300
C18—C19	1.391 (3)	C33—H33	0.9300
C18—C23	1.388 (3)	C34—H34A	0.9600
C19—C20	1.391 (3)	C34—H34B	0.9600
C20—C21	1.386 (3)	C34—H34C	0.9600
C21—C22	1.376 (3)		
			
O2—Cu—O4	173.62 (6)	O4—C25—C26	118.72 (19)
O2—Cu—N1	86.83 (7)	C24—C25—C26	117.77 (19)
O2—Cu—N3	96.06 (7)	C25—C26—C27	121.3 (2)
O2—Cu—O2^i^	81.69 (5)	C26—C27—C28	122.0 (2)
O4—Cu—N1	90.67 (7)	C27—C28—C29	118.7 (2)
O4—Cu—N3	85.87 (7)	C27—C28—C33	121.8 (2)
O2^i^—Cu—O4	104.51 (6)	C29—C28—C33	119.5 (2)
N1—Cu—N3	173.71 (6)	C24—C29—C28	119.0 (2)
O2^i^—Cu—N1	98.82 (6)	C24—C29—C30	122.6 (2)
O2^i^—Cu—N3	87.14 (6)	C28—C29—C30	118.4 (2)
C3—O1—C17	117.92 (19)	C29—C30—C31	120.7 (2)
Cu—O2—C8	118.96 (13)	C30—C31—C32	121.0 (2)
Cu—O2—Cu^i^	98.31 (6)	C31—C32—C33	119.8 (2)
Cu^i^—O2—C8	129.90 (13)	C28—C33—C32	120.7 (2)
C20—O3—C34	117.68 (18)	C1—C2—H6	121.00
Cu—O4—C25	120.22 (14)	C3—C2—H6	121.00
Cu—N1—N2	124.93 (13)	C3—C4—H4	120.00
Cu—N1—C1	121.87 (14)	C5—C4—H4	120.00
N2—N1—C1	111.97 (17)	C4—C5—H3	120.00
N1—N2—C7	121.70 (17)	C6—C5—H3	120.00
Cu—N3—N4	122.90 (13)	C1—C6—H2	121.00
Cu—N3—C18	124.34 (14)	C5—C6—H2	121.00
N4—N3—C18	111.64 (17)	C8—C9—H9	119.00
N3—N4—C24	121.91 (18)	C10—C9—H9	119.00
N1—C1—C2	119.78 (19)	C9—C10—H10	119.00
N1—C1—C6	118.75 (19)	C11—C10—H10	119.00
C2—C1—C6	121.5 (2)	C12—C13—H13	120.00
C1—C2—C3	118.8 (2)	C14—C13—H13	120.00
O1—C3—C2	123.5 (2)	C13—C14—H14	120.00
O1—C3—C4	116.4 (2)	C15—C14—H14	120.00
C2—C3—C4	120.1 (2)	C14—C15—H15	120.00
C3—C4—C5	120.3 (2)	C16—C15—H15	120.00
C4—C5—C6	120.7 (2)	C11—C16—H16	119.00
C1—C6—C5	118.7 (2)	C15—C16—H16	120.00
N2—C7—C8	124.5 (2)	O1—C17—H17A	109.00
N2—C7—C12	114.30 (19)	O1—C17—H17B	109.00
C8—C7—C12	121.2 (2)	O1—C17—H17C	109.00
O2—C8—C7	123.5 (2)	H17A—C17—H17B	110.00
O2—C8—C9	119.14 (19)	H17A—C17—H17C	109.00
C7—C8—C9	117.3 (2)	H17B—C17—H17C	109.00
C8—C9—C10	121.6 (2)	C18—C19—H19	120.00
C9—C10—C11	122.3 (2)	C20—C19—H19	120.00
C10—C11—C12	118.5 (2)	C20—C21—H21	120.00
C10—C11—C16	122.0 (2)	C22—C21—H21	120.00
C12—C11—C16	119.5 (2)	C21—C22—H22	120.00
C7—C12—C11	118.9 (2)	C23—C22—H22	120.00
C7—C12—C13	123.0 (2)	C18—C23—H23	121.00
C11—C12—C13	118.1 (2)	C22—C23—H23	121.00
C12—C13—C14	120.8 (2)	C25—C26—H26	119.00
C13—C14—C15	120.6 (3)	C27—C26—H26	119.00
C14—C15—C16	120.0 (3)	C26—C27—H27	119.00
C11—C16—C15	121.0 (2)	C28—C27—H27	119.00
N3—C18—C19	120.37 (19)	C29—C30—H30	120.00
N3—C18—C23	118.57 (19)	C31—C30—H30	120.00
C19—C18—C23	121.1 (2)	C30—C31—H31	120.00
C18—C19—C20	119.0 (2)	C32—C31—H31	120.00
O3—C20—C19	123.6 (2)	C31—C32—H32	120.00
O3—C20—C21	116.0 (2)	C33—C32—H32	120.00
C19—C20—C21	120.4 (2)	C28—C33—H33	120.00
C20—C21—C22	120.1 (2)	C32—C33—H33	120.00
C21—C22—C23	120.7 (2)	O3—C34—H34A	109.00
C18—C23—C22	118.8 (2)	O3—C34—H34B	109.00
N4—C24—C25	123.76 (19)	O3—C34—H34C	109.00
N4—C24—C29	115.09 (19)	H34A—C34—H34B	110.00
C25—C24—C29	120.95 (19)	H34A—C34—H34C	109.00
O4—C25—C24	123.49 (19)	H34B—C34—H34C	110.00
			
N1—Cu—O2—C8	−45.86 (15)	C2—C3—C4—C5	−0.7 (4)
N1—Cu—O2—Cu^i^	99.40 (6)	C3—C4—C5—C6	−0.4 (4)
N3—Cu—O2—C8	128.53 (15)	C4—C5—C6—C1	0.9 (3)
N3—Cu—O2—Cu^i^	−86.20 (6)	N2—C7—C8—O2	−0.1 (4)
O2^i^—Cu—O2—C8	−145.26 (15)	N2—C7—C8—C9	−179.0 (2)
O2^i^—Cu—O2—Cu^i^	0.00 (5)	C12—C7—C8—O2	178.4 (2)
N1—Cu—O4—C25	−137.83 (16)	C12—C7—C8—C9	−0.5 (3)
N3—Cu—O4—C25	47.43 (16)	N2—C7—C12—C11	−178.1 (2)
O2^i^—Cu—O4—C25	−38.53 (16)	N2—C7—C12—C13	0.5 (3)
O2—Cu—N1—N2	34.04 (15)	C8—C7—C12—C11	3.2 (3)
O2—Cu—N1—C1	−159.66 (15)	C8—C7—C12—C13	−178.1 (2)
O4—Cu—N1—N2	−140.09 (15)	O2—C8—C9—C10	178.6 (2)
O4—Cu—N1—C1	26.21 (15)	C7—C8—C9—C10	−2.4 (3)
O2^i^—Cu—N1—N2	115.12 (15)	C8—C9—C10—C11	2.6 (4)
O2^i^—Cu—N1—C1	−78.58 (15)	C9—C10—C11—C12	0.4 (3)
O2—Cu—N3—N4	146.18 (15)	C9—C10—C11—C16	−179.2 (2)
O2—Cu—N3—C18	−20.73 (16)	C10—C11—C12—C7	−3.2 (3)
O4—Cu—N3—N4	−39.93 (15)	C10—C11—C12—C13	178.1 (2)
O4—Cu—N3—C18	153.17 (16)	C16—C11—C12—C7	176.4 (2)
O2^i^—Cu—N3—N4	64.85 (15)	C16—C11—C12—C13	−2.3 (3)
O2^i^—Cu—N3—C18	−102.06 (16)	C10—C11—C16—C15	−178.7 (2)
O2—Cu—O2^i^—Cu^i^	0.00 (6)	C12—C11—C16—C15	1.8 (4)
O2—Cu—O2^i^—C8^i^	−139.47 (17)	C7—C12—C13—C14	−177.6 (2)
O4—Cu—O2^i^—Cu^i^	−178.46 (6)	C11—C12—C13—C14	1.0 (4)
O4—Cu—O2^i^—C8^i^	42.07 (17)	C12—C13—C14—C15	1.0 (4)
N1—Cu—O2^i^—Cu^i^	−85.46 (7)	C13—C14—C15—C16	−1.6 (4)
N1—Cu—O2^i^—C8^i^	135.08 (17)	C14—C15—C16—C11	0.2 (4)
N3—Cu—O2^i^—Cu^i^	96.56 (7)	N3—C18—C19—C20	−178.9 (2)
N3—Cu—O2^i^—C8^i^	−42.91 (17)	C23—C18—C19—C20	1.1 (3)
C17—O1—C3—C2	−5.9 (3)	N3—C18—C23—C22	178.8 (2)
C17—O1—C3—C4	175.1 (2)	C19—C18—C23—C22	−1.3 (3)
Cu—O2—C8—C7	38.8 (3)	C18—C19—C20—O3	179.4 (2)
Cu—O2—C8—C9	−142.38 (17)	C18—C19—C20—C21	0.1 (3)
Cu^i^—O2—C8—C7	−93.9 (2)	O3—C20—C21—C22	179.5 (2)
Cu^i^—O2—C8—C9	84.9 (2)	C19—C20—C21—C22	−1.1 (3)
C34—O3—C20—C19	−4.5 (3)	C20—C21—C22—C23	1.0 (3)
C34—O3—C20—C21	174.8 (2)	C21—C22—C23—C18	0.2 (3)
Cu—O4—C25—C24	−35.0 (3)	N4—C24—C25—O4	−6.0 (3)
Cu—O4—C25—C26	146.85 (17)	N4—C24—C25—C26	172.24 (19)
Cu—N1—N2—C7	−10.0 (3)	C29—C24—C25—O4	179.5 (2)
C1—N1—N2—C7	−177.49 (18)	C29—C24—C25—C26	−2.3 (3)
Cu—N1—C1—C2	−141.16 (17)	N4—C24—C29—C28	−176.91 (19)
Cu—N1—C1—C6	37.2 (3)	N4—C24—C29—C30	4.1 (3)
N2—N1—C1—C2	26.8 (3)	C25—C24—C29—C28	−1.9 (3)
N2—N1—C1—C6	−154.92 (19)	C25—C24—C29—C30	179.0 (2)
N1—N2—C7—C8	−15.3 (3)	O4—C25—C26—C27	−176.6 (2)
N1—N2—C7—C12	166.09 (18)	C24—C25—C26—C27	5.1 (3)
Cu—N3—N4—C24	16.2 (2)	C25—C26—C27—C28	−3.5 (4)
C18—N3—N4—C24	−175.38 (17)	C26—C27—C28—C29	−0.9 (3)
Cu—N3—C18—C19	146.59 (17)	C26—C27—C28—C33	179.5 (2)
Cu—N3—C18—C23	−33.5 (3)	C27—C28—C29—C24	3.6 (3)
N4—N3—C18—C19	−21.6 (3)	C27—C28—C29—C30	−177.4 (2)
N4—N3—C18—C23	158.35 (19)	C33—C28—C29—C24	−176.9 (2)
N3—N4—C24—C25	15.1 (3)	C33—C28—C29—C30	2.2 (3)
N3—N4—C24—C29	−170.09 (17)	C27—C28—C33—C32	177.1 (2)
N1—C1—C2—C3	177.8 (2)	C29—C28—C33—C32	−2.5 (3)
C6—C1—C2—C3	−0.5 (3)	C24—C29—C30—C31	178.9 (2)
N1—C1—C6—C5	−178.8 (2)	C28—C29—C30—C31	−0.1 (3)
C2—C1—C6—C5	−0.5 (3)	C29—C30—C31—C32	−1.7 (4)
C1—C2—C3—O1	−177.9 (2)	C30—C31—C32—C33	1.5 (4)
C1—C2—C3—C4	1.1 (3)	C31—C32—C33—C28	0.7 (4)
O1—C3—C4—C5	178.4 (2)		

Symmetry code: (i) −*x*+1, −*y*, −*z*+2.

### Hydrogen-bond geometry (Å, º)

**Table:**

*D*—H···*A*	*D*—H	H···*A*	*D*···*A*	*D*—H···*A*
C22—H22···O4^ii^	0.93	2.45	3.267 (3)	146
C32—H32···O1^iii^	0.93	2.51	3.394 (4)	158

Symmetry codes: (ii) −*x*+1, −*y*+1, −*z*+2; (iii) −*x*+1/2, *y*−1/2, −*z*+3/2.
