# Supplementary figures and images for: Crystal structure of bis­{μ-1-[(E)-(3-meth­oxy­phen­yl)diazen­yl]naphthalen-2-olato-κ3 N 2,O:O}bis­({1-[(E)-(3-meth­oxy­phen­yl)diazen­yl]naphthalen-2-olato-κ2 N 2,O}copper(II))
Source: Acta Crystallogr E Crystallogr Commun. 2015 Nov 7;71(Pt 12):m211–2. doi: 10.1107/S2056989015020824 (PMC4719838; doi:10.1107/S2056989015020824)

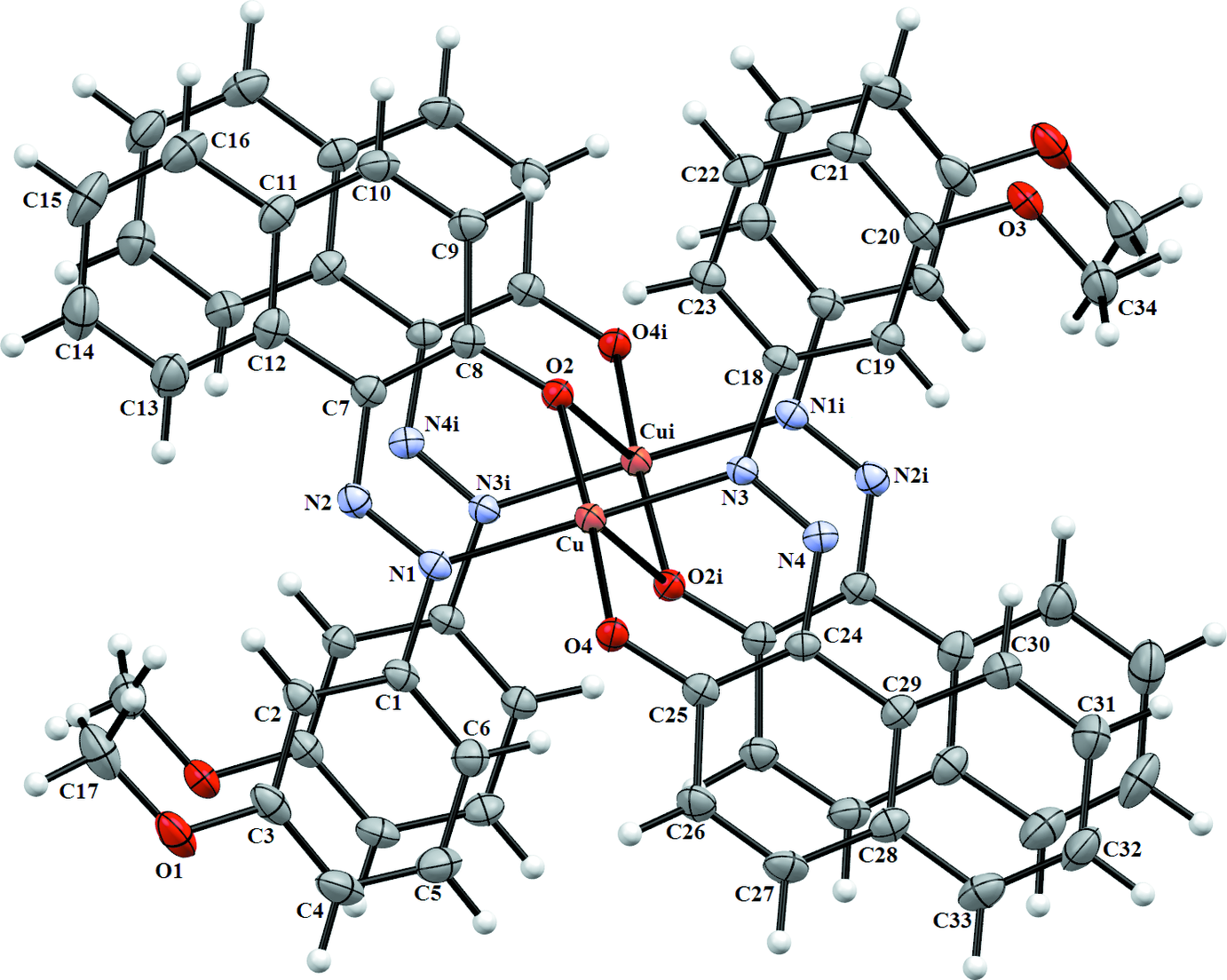

Supplement: Supplementary file 3 [file e-71-0m211-fig1.tif]

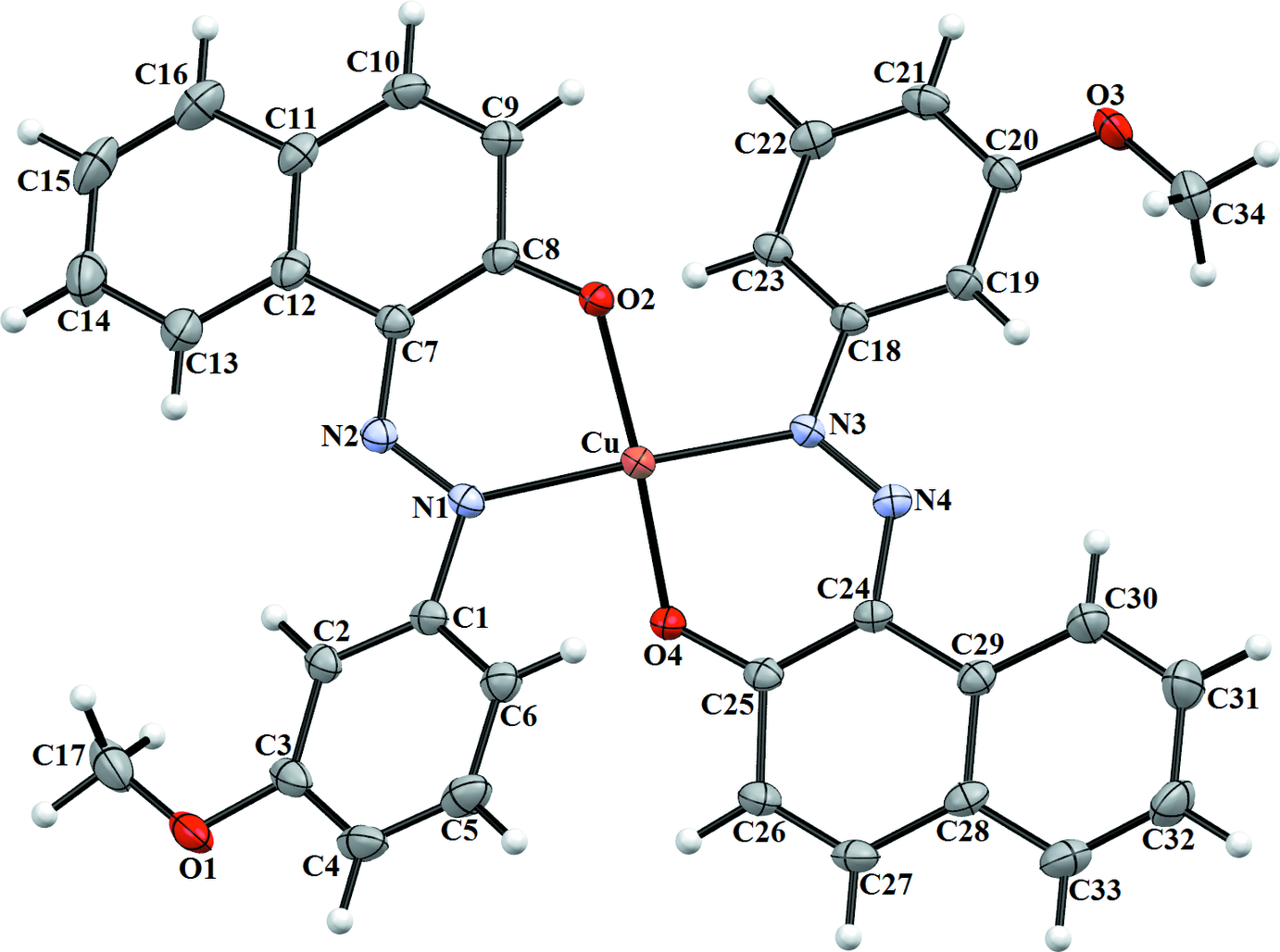

Supplement: Supplementary file 4 [file e-71-0m211-fig2.tif]

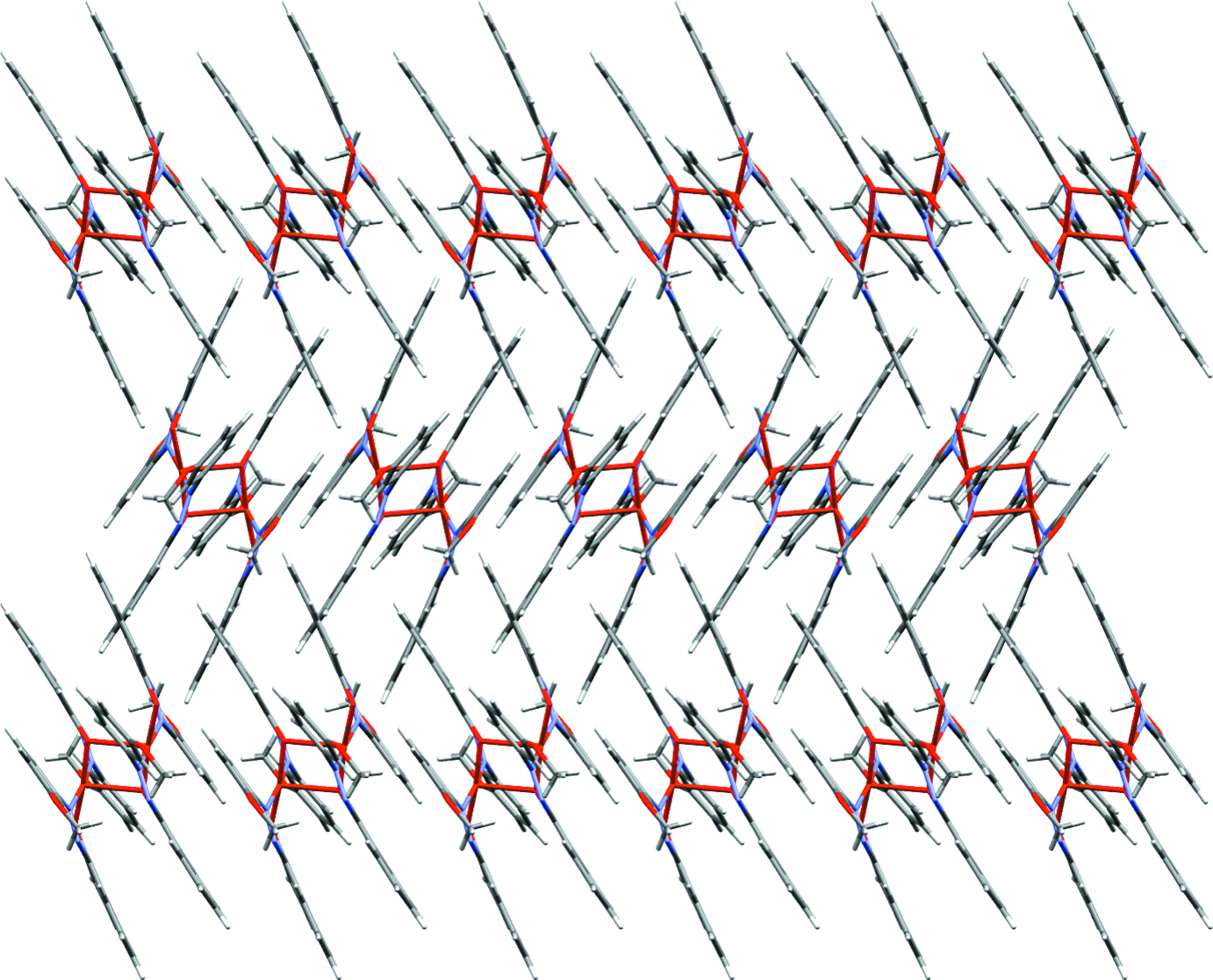

Supplement: Supplementary file 5 [file e-71-0m211-fig3.tif]
